# Relationship between personal recovery, autobiographical memory, and clinical recovery in people with mental illness in the acute phase

**DOI:** 10.1016/j.heliyon.2024.e26075

**Published:** 2024-02-13

**Authors:** Taisuke Mori, Ritsuko Hattori, Keisuke Irie, Kosuke Tsurumi, Toshiya Murai, Ryouhei Ishii, Hiroyuki Inadomi

**Affiliations:** a Department of Day Care Unit, Kyoto University Hospital, Japan; b Osaka Prefecture University Graduate School of Comprehensive Rehabilitation, Japan; c Kyoto University Graduate School of Medicine, Japan; d Osaka Metropolitan University Graduate School of Rehabilitation Science, Japan

**Keywords:** Personal recovery, Autobiographical memory, Clinical recovery, Mental illness

## Abstract

**Aim:**

Narratives are important in psychiatric rehabilitation. People with a psychiatric diagnosis find it difficult to recall specific autobiographical memories of events that lasted less than a day. Although personal narratives play a central role in personal recovery, the factors influencing personal and clinical recovery, such as psychiatric symptoms and cognitive function, have not been fully explored. Therefore, this study examined the associations between personal recovery and autobiographical memory, age, psychiatric symptoms, and neurocognitive function.

**Method:**

The Self-Identified Stage of Recovery, Parts A and B (SISR-A, SISR-B), Autobiographical Memory Test (AMT), Japanese version of the Brief Assessment Scale of Schizophrenia Cognitive Function, and Brief Psychiatric Symptom Rating Scale were administered to 40 individuals with psychiatric disorders who were undergoing psychiatric rehabilitation.

**Results:**

A significant positive correlation was found between the total number of specific memories in the AMT and total SISR-B scores. A binary logistic regression analysis revealed that the total number of specific memories, especially high responsiveness to negative cue words, significantly predicted greater personal recovery. Age, psychiatric symptoms, and neurocognitive function did not significantly predict higher personal recovery.

**Conclusion:**

In psychiatric rehabilitation, negative episodes should be treated with caution; however, they may also facilitate personal recovery.

## Highlights

• Higher autobiographical memory specificity predicts greater personal recovery.
• Those with high personal recovery were able to narrate their negative memories.
• Integrating negative episodes may facilitate personal recovery.

## 1 Introduction

For people with mental disorders, talking about their life experiences promotes psychological growth, including the formation and reintegration of identities [1], maintenance and strengthening of interpersonal relationships [2], and construction of a positive future [3]. Previous studies have reported that recalling positive autobiographical memories results in suppressing cortisol elevation and decreasing negative emotions [4]. Life review therapy, which involves attempts to recall autobiographical memories with accuracy, is aimed at reviewing positive life events [5]. In recent years, psychiatric rehabilitation has tended to focus on the recall of positive memories. However, people with mental illnesses are more likely to recall and process negative memories [6]. Negative memories have maladaptive consequences such as inability to take on new challenges [7], and therapists encourage patients to reinterpret the meaning of the negative event [8]. Nonetheless, negative memory functions both adaptively and maladaptively, and people tend to retain the lessons learnt from the event even if it is a bitter memory [7]. Thus, focusing on negative memories is valuable, and treatment that enables people with mental illness to discuss their life experiences has important implications for psychiatric rehabilitation.

As a common clinical phenomenon, it is difficult for people with mental illnesses to recall specific autobiographical memories of events that lasted less than a day [9]. In contrast, patients with recalling difficulties, such as patients with various mental illnesses, are able to recall common events that either occur multiple times or over a long period [9]. This phenomenon is termed “reduced autobiographical memory specificity.” The Autobiographical Memory Test (AMT; Williams & Broadbent [10]) is the most common method for quantitatively assessing the specificity of autobiographical memory [9]. In the AMT, a subject is given cue words and asked to report a past event for each word. Research suggests that certain autobiographical memories are not forgotten but are difficult to retrieve [9]. A recent meta-analysis indicated a wide existence of reduced autobiographical memory specificity in psychiatric disorders, with no differences across diagnostic groups [9]. In other words, reduced autobiographical memory specificity is a commonly observed phenomenon in patients with mental illness.

Shiers et al. [11] suggested that personal narratives that enhance the quality of life may restore individuality. These personal narratives play a central role in recovery under the guidance of psychiatry, particularly within the framework of personal recovery [12]. The relationship between personal narratives and recovery has been reported in schizophrenia [13]. After controlling for psychiatric symptoms, self-esteem, hope, and vocabulary, the study suggests that an enhanced personal narrative may lead to the development of relationships with others and improved functioning in the community. Thus, personal narratives are often discussed in the recovery literature, but there are not many studies have examined the relationship between recovery and the ability to tell stories. Furthermore, psychiatric rehabilitation interventions target various mental illnesses, but the relationship between autobiographical memory and personal recovery in mental illnesses, including schizophrenia, has not been sufficiently investigated.

Some people feel that mental illness compromises their lives, as even after improvement in symptoms with treatment, various life difficulties remain. Recovery is defined as “the process in which people are able to live, work, learn, and participate fully in their communities. For some individuals, recovery is the ability to live a fulfilling and productive life despite a disability. For others, recovery implies the reduction or complete remission of symptoms [14].” The concept of recovery is applicable to many individuals with mental illnesses other than schizophrenia [[15], [16], [17], [18]]. Recovery is broadly classified into clinical recovery (i.e., the objective improvement in symptoms and functioning) and personal recovery (i.e., the restoration of self-worth and meaningful life) [19].

Personal recovery is a way to live a life of contentment, hope, and contribution despite the limitations caused by illnesses [20]. The main components of personal recovery are connectedness, hope and optimism about the future, identity, meaning in life, and empowerment [21]. Previous studies have reported several factors that influence personal recovery. Personal recovery rate was found to be higher in younger age groups [22,23]. Conversely, older individuals have been reported to be less responsive to interventions for personal recovery [24]. Moreover, low personal recovery is associated with greater severity of psychiatric symptoms, both in general as well as in each domain, including positive, negative [25], affective [26], and depressive [27,28] symptoms. Regarding neurocognitive function, domains such as visual memory, working memory, attention [29], and executive function [30] may be related to personal recovery. However, most previous studies on recovery have only explored the subject's own story to examine how narratives change [31]. Additionally, factors such as psychiatric symptoms and neurocognitive functioning have been explored individually, and factors that influence personal recovery, including the specificity of autobiographical memory, remain unexplored, and their impact has not yet been quantitatively examined.

Thus, we hypothesized that autobiographical memory—especially due to its high responsiveness to positive and negative cue words—may contribute to personal recovery in individuals with mental illness. In this study, we primarily examined the relationship between personal recovery and autobiographical memory, which is considered to affect personal recovery in people with mental illness undergoing psychiatric rehabilitation (psychiatric occupational therapy). We also comprehensively examined the relationships among age, psychiatric symptoms, and neurocognitive functioning, which are known to affect personal recovery.

## 2 Methods

### 2.1 Participants and procedures

The participants were selected from among those who were prescribed psychiatric occupational therapy during their stay at a university hospital and scored ranging from 51 to 67 on the Japanese version of the modified Global Assessment of Functioning Scale (mGAF [32]). The mGAF is a numerical scale ranging 1–100 that is used to assess social, occupational, and psychological functioning; a score of 51–67 indicates persistent minor to moderate problems with social, occupational, or school functioning. The participants were 40 inpatients (10 men and 30 women) diagnosed with schizophrenia, bipolar disorder, depression, autism spectrum disorder, and eating disorders by psychiatrists. The exclusion criteria were persons with dementia or traumatic brain injury who were judged by psychiatrists as difficult to understand or unable to participate. The Medical Ethics Committee of the authors’ hospital approved this study. All participants who met the inclusion criteria were informed about the experiments, and only those who provided signed informed consent were included.

### 2.2 Measures

#### 2.2.1 Autobiographical Memory Test

Autobiographical memory was assessed using the AMT [10], which requires subjects to recall specific memories in response to cue words. The cue words consisted of positive (e.g., happy, safe, interested, successful, and surprised) and negative (e.g., sorry, angry, clumsy, hurt, and lonely) words, following Williams and Broadbent's [10] study and ten cue words were presented with alternating positive and negative words. The time limit for each cue word was 60 s, and if participants could not recall anything within this time, they proceeded to the next cue word. The recalled memories were classified into five categories. Specific memory refers to a personal past experience that occurred at a specific time and place and lasted no longer than a day (e.g., “Five years ago, I was happy at my wedding”). An extended memory is of an event that lasted longer than a day (e.g., “I went to the U.S. for a summer vacation”). Categoric memories are those of an aggregated recurring event (e.g., “the train was crowded every morning”). Semantic association refers to cases in which the subject reports semantically related information and not specific memories, especially connotations about people or things (e.g., “I was lonely”). If a memory did not fall into any of these four categories or did not emerge within the time limit, it was classified as an omission. The AMT score was calculated as the total number of specific memories and the number of specific memories for each positive and negative cue words (positive specific memories, negative specific memories). In this study, the author conducted the interviews in person. The author confirmed the AMT instructions, administered them to several healthy subjects, and then interviewed the participants.

#### 2.2.2 Self-Identified Stage of Recovery, Parts A and B

Personal recovery was assessed using the Self-Identified Stage of Recovery, Parts A and B (SISR-A, SISR-B [33]). The SISR-A assesses the stage of recovery by asking participants to choose the most applicable of five sentences that describe the moratorium, awareness, preparation, rebuilding, and growth stages. The SISR-B comprises four items on finding hope, reestablishment of identity, finding meaning, and taking responsibility—the main components of the recovery process. These items are scored on a Likert scale ranging from strongly disagree (1) to strongly agree (6), with higher total scores indicating greater recovery. In Japan, reliability and validity have been reported for patients with mental illness [33]. In the present study, the SISR-B scores were set according to the mean scores of the SISR-B in Chiba et al.’s [33] study, which examined people with mental illness in the community and inpatients in Japan, as follows: high recovery group ≥16 and low recovery group ≤15.

#### 2.2.3 Japanese version of the Brief Assessment of Cognition in Schizophrenia

The Japanese version [34] of the Brief Assessment of Cognition in Schizophrenia (BACS-J [35]) was used to assess neurocognitive function. Six subtests were used to assess verbal memory and learning, working memory, motor speed, verbal fluency, attention and information processing speed, executive function, and the overall cognitive level (composite score)—Verbal Memory, Digit Sequencing, Token Motor Task, Verbal Fluency, Symbol Coding, and Tower of London. The composite score for each subtest was converted to a Z-score, with a higher Z-score indicating higher neurocognitive function. The reliability and validity of the Japanese version have been reported previously [34].

#### 2.2.4 Brief Psychiatric Rating Scale

The Japanese translation of the Oxford version of the Brief Psychiatric Rating Scale (BPRS; [36,37]) was used to assess psychiatric symptoms. The BPRS consists of 18 symptom items rated on a scale ranging from 0 (no symptoms) to 6 (very severe). Higher total scores indicate greater severity of psychiatric symptoms.

### 2.3 Data analysis

The normality of the distribution of basic information was confirmed using the Shapiro–Wilk test. Next, the total SISR-B scores were classified into the following two groups: “high recovery” and “low recovery.” Multicollinearity was confirmed through correlation analysis using Spearman's rank correlation coefficient. Binary logistic regression analysis (forced entry method) was used to analyze the associations between the levels of personal recovery and autobiographical memory, age, psychiatric symptoms, and neurocognitive function, with the binary variables of high and low recovery as the dependent variables. We investigated predictive factors by adding autobiographical memory to the age, BPRS scores, and BACS-J composite scores, which were suggested to be related as independent variables in previous studies. We used SPSS 28.0 statical software to perform the data analysis. Logistic regression analysis was performed using the JMP Pro 16.0, with the significance level set at p < .05. The sample size was calculated based on the number of independent variables (10 times the number of independent variables) to be entered into binomial logistic regression analysis [38]. As there were four independent variables, the sample size was set to 40.

## 3 Results

The study included 40 individuals, with a majority being female (30/40, 75%). The demographic and clinical characteristics of patients are shown in Table 1. The values of each measure were compared by sex, and no significant differences were found in any of the variables. Bivariate correlations revealed a significant positive correlation between the total number of specific memories in the AMT and SISR-B scores. In particular, a significant association was found between the number of negative specific memories and total SISR-B scores. However, there was no significant association between the number of positive specific memories and total SISR-B scores (Table 2). The items that showed multicollinearity with total SISR-B scores of 0.8 or higher were reestablishment of identity and finding meaning, which were reduced to the SISR-B score. Age, psychiatric symptoms, and neurocognitive function were not significantly associated with personal recovery.

**Table 1:** Participants’ demographic and clinical characteristics.

	Means ± Standard Deviation
Age (years)	39.6 ± 14.9
Age of onset of illness (years)	26.1 ± 11.1
Duration of illness (years)	13.5 ± 9.6
Education (years)	14.0 ± 2.3
mGAF	59.9 ± 4.1
AMT	
total specific memories (0-10)	5.9 ± 2.1
positive specific memories (0–5)	3.1 ± 1.2
negative specific memories (0–5)	2.8 ± 1.3
extended (0-10)	1.0 ± 1.0
categorical (0-10)	2.0 ± 1.7
semantic association (0-10)	0.4 ± 0.6
omissions (0-10)	0.8 ± 1.1
SISR	
Part A(1–5)	3.0 ± 1.1
Part B	
B-1(1–6)	3.2 ± 1.3
B-2(1–6)	3.6 ± 1.3
B-3(1–6)	3.4 ± 1.5
B-4(1–6)	4.0 ± 1.5
total score (4-24)	14.2 ± 4.6
BACS-J Composite score	−0.9 ± 1.4
BPRS total (0–108)	9.4 ± 5.8

mGAF: Japanese version of the modified Global Assessment of Functioning scale.

AMT: Autobiographical memory test.

SISR: Self-Identified Stage of Recovery.

BACS-J: Japanese version of the Brief Assessment of Cognition in Schizophrenia.

BPRS: Brief Psychiatric Rating Scale.

**Table 2:** Intercorrelations of the variables.

		SISR											AMT				
Variable		B-1	B-2	B-3	B-4	total	SISR-A	Age	Age of onset of illness	Duration of illness	Education	mGAF	Total specific memories	Positive specific memories	Negative specific memories	BACS-J composite score	BPRS total
SISR	B-1		.470**	.595**	.502**	.775**	.250	−.057	.064	−.119	−.037	−.066	.315*	.132	.386*	−.097	.213
	B-2			.695**	.522**	.809**	.237	.007	.130	−.033	−.074	.043	.237	.023	.330*	−.043	.045
	B-3				.452**	.857**	.219	−.107	.143	−.254	−.096	.076	.317*	.265	.279	.063	.001
	B-4					.777**	.387*	−.028	.143	−.132	−.075	.023	.204	.124	.184	−.179	.034
	total						.360*	−.062	.150	−.180	−.102	.021	.338*	.198	.346*	−.087	.116

Spearman's rank correlation coefficient *p < .05，**p < .01.

mGAF: Japanese version of the modified Global Assessment of Functioning scale.

AMT: Autobiographical Memory Test.

SISR: Self-Identified Stage of Recovery.

B-1: finding hope, B-2: reestablishment of identity, B-3: finding meaning, B-4: taking responsibility.

BACS-J: Japanese version of the Brief Assessment of Cognition in Schizophrenia.

BPRS: Brief Psychiatric Rating Scale.

Binomial logistic regression analysis was performed to examine variables affecting high personal recovery among autobiographical memory, age, psychiatric symptoms, and neurocognitive functioning (Table 3). The total number of specific memories in the AMT was an independent predictor of greater personal recovery (odds ratio [OR] 1.736, 95% confidence interval [CI] 1.077–2.797, p < .05). In particular, the number of specific memories for negative cue words was found to significantly predict personal recovery (OR 2.245, 95% CI 1.044–4.831, p < .05).

**Table 3:** Logistic regression analyses for the variables predicting personal recovery.

Variable	β	Exp(B)	Exp(B) 95%CI		p
			LL	UL	
AMT(the total number of specific memories)	0.552	1.736	1.077	2.797	0.023*
age	0.003	1.003	0.945	1.065	0.922
BPRS	0.056	1.057	0.912	1.226	0.459
BACS-J	−0.417	0.659	0.330	1.316	0.237
					
AMT(the total number of negative specific memories)	0.809	2.245	1.044	4.831	0.039*
age	−0.010	0.581	0.935	1.048	0.727
BPRS	0.030	1.031	0.884	1.203	0.700
BACS-J	−0.402	0.669	0.344	1.302	0.237
					
AMT(the total number of positive specific memories)	0.685	1.984	0.972	4.048	0.060
age	0.005	1.005	0.949	1.065	0.856
BPRS	0.102	1.107	0.963	1.272	0.153
BACS-J	−0.246	0.782	0.424	1.441	0.430

a Autobiographical Memory Test.

b Brief Psychiatric Rating Scale.

c Japanese version of the Brief Assessment of Cognition in Schizophrenia.

## 4 Discussion

We examined the associations between personal recovery and autobiographical memory, age, psychiatric symptoms, and neurocognitive functioning. The results showed that a higher specificity of autobiographical memory was an independent predictor of greater personal recovery in individuals with mental illness. However, age [[22], [23], [24]], psychiatric symptoms [[25], [26], [27], [28]], and neurocognitive function [29,30], which were suggested to be associated in previous studies, were not associated with personal recovery in the present study. Most previous studies on personal recovery involved community-dwelling outpatients in specific disease groups and not inpatients or those with various mental illnesses. However, most participants in the current study were hospitalized patients with relatively mild psychiatric symptoms who were participating in psychiatric rehabilitation. Decreased autonomy and decision-making in the inpatient setting have been noted in the past [39], and it is possible that the participants in this study were more controlled in terms of their environment. Along with the psychopathological heterogeneity of the participants, this environmental specificity may explain the difference between our results and previous findings.

A large number of specific memories in the AMT predicts greater personal recovery. Any memory has the potential to function adaptively [7], and these memories may help subjects find meaning in the present and hope for the future, as suggested in previous research [40] on autobiographical memory functioning and higher well-being. Autobiographical memory is considered to have the following three functions: first, the self-function supports self-consistency and self-identity; second, the social function facilitates interpersonal communication; third, the directive function uses the past to guide present and future thoughts and behaviors [41]. The first and second functions of autobiographical memory conceptually overlap with the two main components of personal recovery—connectedness and identity—respectively. This may explain the association between autobiographical memory and personal recovery observed in our study.

In the present study, we used the number of specific memories for each of the positive and negative cue words in the AMT and found that only higher responsiveness to negative cue words was associated with personal recovery. Narrative interventions focus on the recall of positive memories [4,5]. Intuitively, recalling positive events rather than negative events is assumed to contribute more to personal recovery. In fact, positive memories have been reported to be helpful in stress coping [4] and problem solving [7], to function adaptively, and to be harmless [7]. However, our results suggest that negative events are important from the perspective of personal recovery. One possible explanation of these findings is that the process of confrontation and re-integration of past negative events into one's own identity may improve subjective well-being and aide personal recovery in the long run [42]. The participants in this study were inpatients. Some subjects were kept in a quiet room immediately after admission, while others were admitted without consent. In addition to being more likely to process negative memories [7], the circumstances leading to hospitalization may have made them feel more isolated during their stay [43]. Despite that situation, participants showing higher personal recovery still vividly remembered the circumstances that led to their hospitalization, but at the same time they may have been thinking about how to prevent something like that from happening again (especially the negative cue words “hurt” and “lonely”). A recent study suggested that focusing on one's own experiences after the onset of mental illness, reviewing negative events, and finding meaning and value may lead to personal recovery [44]. Furthermore, personal negative memories serve as lessons and assist everyday decision-making by applying those lessons to current problems [45]. However, random recall of negative memories, including traumatic events, may have adverse effects. In psychiatric rehabilitation, negative memories should be handled with caution. In this study, as the subjects were asked to recall their memories in a semi-structured fashion using the AMT, we suspect that the majorities of the recalled memories were those that have already been re-integrated in the individual's personal history. We speculate that this is the reason why negative recall was found to be correlated with personal recovery in our study.

### 4.1 Limitation and strength

This study had some limitations. First, the sample size was small. Although this study included patients with several mental illnesses, it was not possible to examine each disorder separately. It is also possible that of the various diseases were being treated with different medications, which may have affected participants’ speech and arousal levels. In addition, there were more women than men in the present study. This was because more women than men were inpatients and participants of psychiatric occupational therapy. In fact, it has been reported that women with emotional problems are more likely to seek psychiatric help than men [46]. Many studies have reported that women are more specific and detailed in their narratives, and more accurate in their recall of events [47]. Although there were no differences in the variables between men and women in this study, it is conceivable that there was an effect on the representation of the narratives. However, as people with various diseases participate in clinical practice psychiatric rehabilitation, the present results may reflect the real clinical practicality of psychiatric rehabilitation. Second, we did not assess rumination and defense mechanisms, included in the hypothesized model [48], which would account for the reduced specificity of autobiographical memory. It may be necessary to distinguish between what can be retrieved and what can be shared with others. In the AMT in this study, participants reported their memories verbally to the experimenter. Barry et al. [9] found that people who were rejected or criticized for sharing certain memories in the past may resist sharing personal information with others. It is possible that some subjects were able to recall specific memories but unable to express them. In addition, the role of metacognitive abilities in the recall of autobiographical memories has recently been reported, which is useful for monitoring memory retrieval and for more detailed and contextually relevant access [49]. Therefore, it is necessary to examine the mechanisms behind the influence of autobiographical memories on personal recovery, including physiological indices. Third, the cross-sectional design of this study limited the significance of the findings. A previous study conducted in Japanese acute care wards that focused on medication [39] found no longitudinal changes in personal recovery. Therefore, the effects of personal narratives shared within psychosocial treatment interventions on personal recovery have not been examined and should be evaluated longitudinally.

Despite these limitations, our study is the first to clarify the direct relationship between autobiographical memory and personal recovery in persons with mental illness while considering the influences of participants’ age and functional aspects. In terms of treatment, our results may provide hints for treating both positive and negative events in a structured manner, rather than randomly recalling autobiographical memories.

## 5 Conclusions

In this study, the total number of specific memories in the AMT was an independent predictor of greater personal recovery. In particular, those with high personal recovery were able to narrate their negative memories. At first glance, talking about happy events appears to be more effective in improving well-being and quality of life. However, an individual can accept their own story by integrating negative events and remembering things that did not go their way in terms of personal recovery. In psychiatric rehabilitation, although negative episodes should be treated with caution, they may facilitate personal recovery.

## Ethics declarations

This study was reviewed and approved by Kyoto University Graduate School and Faculty of Medicine, Ethics Committee, with the approval number: [R2819].

## Funding statement

This work was supported by 10.13039/501100001691JSPS KAKENHI [Grant Number 21H04320].

## Data availability statement

Data will be made available on request.

## CRediT authorship contribution statement

**Taisuke Mori:** Writing – original draft, Visualization, Software, Resources, Methodology, Investigation, Funding acquisition, Formal analysis, Data curation, Conceptualization. **Ritsuko Hattori:** Investigation, Data curation. **Keisuke Irie:** Investigation, Data curation, Conceptualization. **Kosuke Tsurumi:** Writing – review & editing, Supervision. **Toshiya Murai:** Writing – review & editing, Supervision, Project administration. **Ryouhei Ishii:** Writing – review & editing, Supervision, Methodology, Conceptualization. **Hiroyuki Inadomi:** Writing – review & editing, Validation, Supervision, Project administration, Methodology, Funding acquisition, Formal analysis, Conceptualization.

## Declaration of competing interest

The authors declare that they have no known competing financial interests or personal relationships that could have appeared to influence the work reported in this paper.
